# BANK1 Regulates IgG Production in a Lupus Model by Controlling TLR7-Dependent STAT1 Activation

**DOI:** 10.1371/journal.pone.0156302

**Published:** 2016-05-26

**Authors:** Ying-Yu Wu, Ramesh Kumar, Ryuji Iida, Harini Bagavant, Marta E. Alarcón-Riquelme

**Affiliations:** 1 Arthritis and Clinical Immunology Program, Oklahoma Medical Research Foundation, Oklahoma City, OK, 73104, United States of America; 2 Department of Medical Genomics, Pfizer/University of Granada/Andalusian Government Center for Genomics and Oncological Research (GENYO), 18016, Parque Tecnológico de la Salud (PTS), Granada, Spain; Karolinska Institutet, SWEDEN

## Abstract

The purpose of our study was to investigate the effects of the adaptor Bank1 in TLR7 signaling using the B6.*Sle1*.*yaa* mouse, a lupus model that develops disease through exacerbated TLR7 expression. Crosses of B6.*Sle1*.*yaa* with *Bank1*^*-/-*^ mice maintained several B and myeloid cell phenotypes close to normal wild-type levels. Most striking was the reduction in total serum IgG antibodies, but not of IgM, and reduced serum levels of autoantibodies, IL-6, and BAFF. *Bank1* deficiency did modify numbers of MZ B cells and total B cell numbers, as well as expression of CXCR4 by follicular helper T cells. Other T cell changes were not observed. *Bank1* deficiency did not modify numbers of germinal center B cells or plasma cells or clinical disease outcomes. Purified B cells from *Bank1* deficient mice had strongly reduced *Ifnb*, *Ifna4*, *Irf7*, *Aicda* and *Stat1* gene expression following TLR7 agonist stimulation. Interestingly, phosphorylation of Tyr701, but not of Ser727 of STAT1, was impaired in splenic B cells from *B6*.*Sle1*.*yaa*.*Bank1-/-* mice, as was the nuclear translocation of IRF7 in response to TLR7 agonist stimulation. Further, *Bank1* deficiency in B6.*Sle1*.*yaa* mice reduced the production of IgG2c after *in vitro* TLR7 agonist stimulation. Our results demonstrate that *Bank1* controls TLR7-mediated type I interferon production. Combined with the control of the nuclear translocation of IRF7, the modulation of STAT1 transcription and phosphorylation, *Bank1* contributes to IgG production during development of autoimmune disease.

## Introduction

B-cell scaffold with ankyrin repeats 1 (BANK1) is an adaptor protein primarily expressed in B cells and to a lesser extent in myeloid cells and plasmacytoid dendritic cells [1, 2]. BANK1 spans 785 amino acids in its full-length, contains repeat motifs and coil-coiled regions, as well as 13 tyrosines susceptible of phosphorylation and interaction with SH2 domains. BANK1 binds to and is phosphorylated by the Src-family of tyrosine kinases LYN and BLK, and interaction with the latter is required for BANK1 to bind phospholipase C gamma 2 (PLCγ2) [3]. The short isoform of BANK1, found in human and mouse, lacks exon 2, which encodes for a conformational Toll-like/IL-1 (TIR) domain [4].

Deficiency of *Bank1* in the mouse has two apparently opposing effects: on the one hand, activation of CD40-induced phosphorylation of Akt and increase of IgM and germinal center formation [2]. On the other hand, a decrease in CpG-induced IL-6 production due to reduced mitogen activated protein kinase (MAPK) p38 phosphorylation leading directly to a specific reduction of the MAP kinase interacting serine/threonine kinases 1 and 2 (Mnk1/2) [5]. This in turn leads to a reduction in the phosphorylation of the translation initiation factor eIF4E. The effect of *Bank1* deficiency on p38 phosphorylation following CpG-induction overrides that induced by CD40 when CpG and anti-CD40 are used simultaneously. CD40 induces a cascade that via mTOR phosphorylates 4E-BP1, a molecule restraining eIF4E that inhibits translation. Thus, release of eIF4E by 4E-BP1 does not avoid the decreased signaling of eIF4E via MNK1/2 when *Bank1* is deficient, with the resultant reduced secretion of IL-6.

Several inflammatory, autoimmune diseases have been genetically associated with the human BANK1 gene in genome-wide association studies, such as systemic lupus erythematosus (SLE)[6], systemic sclerosis and rheumatoid arthritis [7–10]. But due to the little information that we have on the function of BANK1, we know even less on how BANK1 is involved in disease pathogenesis.

TLR7, as TLR9, uses leucine rich repeats in the ectodomain to bind their ligands. Ligand binding leads to conformational changes believed to affect the cytoplasmic Toll-like/IL-1 (TIR) domains eliciting signals. Such signals entail the association of TIR domains of TLR7 with the adaptor molecule myeloid differentiation primary response gene 88 (MyD88). In turn, MyD88 interacts via its N-terminal death domain (DD) with IL-1R-associated kinase-4 (IRAK4). IRAK4 phosphorylates IRAK1 and IRAK2. These activate TNF receptor-associated factor 6 (TRAF6). In B cells, macrophages and dendritic cells (DCs), a TRAF6-dependent signaling activation takes place via the mitogen-activated protein kinase kinase (MAP3K-MKK) cascades. MAPKs ERK, JNK and p38 become activated and induce the transcription factor AP-1. Also through the MAP3K involvement, the conserved helix-loop-helix ubiquitous kinase (IKKα/β) becomes activated leading to the phosphorylation and degradation of nuclear factor of kappa light polypeptide inhibitor (*Nfkbia*, also known as IκB) with the resultant activation of the nuclear factor kappa b (NFκB). On another path, MyD88 and TRAF6 associate and activate the transcription factor interferon regulatory factor 7 (IRF7) directly. Activated IRF7 translocate to the nucleus to act on the promoters and subsequently on the transcription of the pro-inflammatory cytokines and type I interferon [10].

Type I interferon production is dependent on the recognition of viral and self nucleic acids by cellular sensors. One such sensor is the endosomal toll-like receptor 7 (TLR7). Deficiency of *Tlr7* leads to complete abrogation of lupus disease in some murine lupus models [11–13] while deficiency of *Tlr9* enhances its severity [11, 12, 14]. The production of type I IFN induced by TLR7 is believed to occur primarily in plasmacytoid dendritic cells, but recently, the role of B cells in the development of lupus dependent on TLR7 was considered essential [15].

Ablation of the IFN alpha receptor (IFNAR) prevents the effective response of B cells to TLR7 stimulation [16]. During TLR7-mediated autoimmunity, when produced by B cells, type I interferon is taken up by IFNAR leading to the transcription of more interferon generating a positive feedback loop [17]. B cells also become receptive to interferon produced by plasmacytoid dendritic cells (pDCs). The engagement of IFN leads to phosphorylation of the signal transducer and activator of transcription 1 (STAT1) and its dimerization leading in turn to the activation of the JAK/STAT cascade of IFN effects [18]. Among the effects induced by TLR7 agonists and augmented by interferon are the generation and class switch recombination of antibodies and autoantibodies [19], the production of pro-inflammatory cytokines and the expression of co-stimulatory molecules in the surface of B cells [17, 20, 21]. In addition, the release of type I interferons leads to a large spectrum of cellular changes and the expression of interferon inducible genes by various hematopoietic cell types including further increase in TLR7 expression [21]. Furthermore, the process is enhanced by co-stimulatory signals, and T-B interactions, leading to the enhanced survival of autoreactive B cells [22].

Among the various lupus models of SLE, the congenic mouse containing the *Sle1* locus, one of three extensive loci identified using the New Zealand-derived recombinant strain NZM2410, is characterized by the presence of several B cell and Dendritic Cell (DC) phenotypes on the C57BL/6 background [23]. The presence of the BXSB-derived *yaa locus*, a duplication of an X chromosome region containing TLR7 translocated to the Y chromosome [24, 25], results in important synergistic effects of the inflammatory and autoimmune responses induced by the genes of the *Sle1* locus in male mice, namely the B6.*Sle1*.*yaa* mice [26, 27].

Recently, Farris and her colleagues reported that a knockout for the IL-6 gene completely abrogated the autoimmune phenotypes of the B6.*Sle1*.*yaa* [28]. Considering our previous results showing that Bank1 controls IL-6 protein secretion in the TLR9 signaling pathway, that Bank1 posses a TIR conformational domain, and to investigate if *Bank1* had any *in vivo* and/or *in vitro* effects on the TLR7 pathway and consequently on the development of lupus and lupus-related phenotypes, we produced crosses of the *Bank1*^*-/-*^ with the B6.*Sle1*.*yaa* strain.

Our results show that BANK1 is involved in TLR7 signaling, support its role as a susceptibility gene for SLE in the human by modulating levels of total IgG and IgG autoantibodies.

## Materials and Methods

### Mice

This study and all funded project that led to the preparation of this manuscript have been approved by the IACUC of the Oklahoma Medical Research Foundation.

*Bank1* KO mice were a generous gift from Dr. T. Kurosaki (RIKEN Institute, Kyoto, Japan). *Bank1* mice were backcrossed 11 generations to C57BL/6J mice. Darise Farris at OMRF generously provided the B6.*Sle1*.*yaa* mice originally obtained from Dr Edward Wakeland, UT Southwestern, Dallas, TX.

Groups of B6.Sle1.yaa.Bank1^*+/+*^ and B6.Sle1.yaa.Bank1^*-/-*^ mice were followed for 36 weeks to monitor mortality with sera collected every 6 weeks starting at 18-wk of age. Some animals were euthanized at 20–24 weeks of age for the study of lupus nephritis, cellular phenotypes and gene expression by RT-PCR. 7~13-wk-old mice were used for B cell signaling analyses.

Wild-type (WT) mice, C57BL/6J were purchased from Jackson Laboratories and used as controls for the flow cytometry experiments. All mice were maintained under specific pathogen-free conditions at the Oklahoma Medical Research Foundation (OMRF).

### Flow cytometry and antibodies

Single-cell suspensions from mouse spleens and bone marrows were incubated with fluorescently labeled antibodies in 10% rabbit serum (Sigma-Aldrich) in FACS buffer (4% FCS+ 0.05% sodium azide in 1X PBS) for 20 minutes at 4°C after blocking nonspecific binding with anti-mCD16/32 (Clone: 2.4G2) or 10% rabbit serum in FACS buffer. Data were acquired on a LSR II flow cytometer (BD) and analyzed using FlowJo 9.8 software (Tree Star).

Antibodies used are: anti–B220-PE-Texas Red (clone RA3-6B2), anti–mCD93-PerCP-Cy5.5 (clone AA4.1), anti–mCD23-eFluor 660 (clone B3B4), anti–mCD21/CD35-PE/Cy7 (clone 7E9), anti–mIgM allophycocyanin/Cy7 (clone RMM-1), anti–mIgD- eFluor 450 (11–26), anti–mCD5-PE (clone 53–7.3), anti-mCD19-APC/Cy7 or APC (clone 6D5), anti-mTCRβ-eFluor 450 (clone H57-597), anti-mCD69-Alex Fluor 647 (clone H1.2F3), anti-mCD4-FITC (clone RM4-5), anti-mCD44-APC (clone IM7), anti-mCD62L-PE/Cy7 (clone MEL-14), anti-PSGL1-PE (clone 2PH1), anti-mCXCR4-V450 (clone 2B11/CXCR4), anti mCXCR5-biotin (clone 2G8), PE/CF594- or PE/TexasRed-Streptavidin, anti-PNA-FITC (Vector, cat# FL-1071), anti-GL7-PE (clone GL-7), anti-mCD3-APC (clone 145-2C11), anti-mCD11c-AF488 (clone N418), anti–mCD138-Brilliant Violet 421 (clone 281–2), anti-mCD11b-FITC (clone M1-70), anti-mCD45.2-PE (clone 104), anti-mLy-6G/Ly-6C (Gr-1)-AF647 (clone RB6-8C5). All antibodies were purchased from BioLegend, eBioscience, or BD Biosciences unless stated otherwise.

### Measurement of serum antibodies, IL-6, BAFF and Blood Urea Nitrogen

For IgG anti-dsDNA ELISAs, 96-well Maxisorp Immuno plates (Nunc) were coated with calf thymus DNA (50 μg/ml) and/or protamine sulphate (Sigma-Aldrich) and were blocked for 1h with 10% calf serum+ 5% goat serum in PBS-T (0.05% Tween 20) prior to addition of diluted serum (1:50 for mouse samples) for 2h. Antibodies were detected using goat anti-mouse IgG-HRP (Southern Biotechnology) and peroxidase reactions were developed using OptEIA TMB substrate (BD). Absorbance at 450 nm was read using a spectrophotometer.

The concentration of total IgM, IgG and IgG2c in serum was quantified using “Mouse IgG ELISA quantitation kit” from eBioscience Inc. and Bethyl Laboratories Inc., respectively. Measurement of IL-6 was done using a “High Sensitivity ELISA Kit with signal amplification” (eBioscience). Sera were diluted 1:10 and experiment was performed in duplicates. Levels of BAFF were quantified using “Mouse BAFF/BLyS/TNFSF13B Quantikine ELISA” kit and manufacturer´s instructions (R&D systems). Sera were diluted 1:24 and the experiment was done in duplicates. O.D. was captured at 450nm absorbance with the correction wavelength set at 570nm.

Blood urea nitrogen (BUN) was measured using QuantiChrom Urea Assay Kit (BioAssay Systems, USA) as per manufacturer’s instructions. Briefly, 24 weeks mice sera were diluted 1:10 in distilled water. Diluted serum, the positive sample and water as a blank were transferred in triplicate into wells of Costar® clear flat bottom 96 well plates (Corning Incorporated, NY USA). A mixture of working reagent was then added and the plate was incubated for 20 min at room temperature; finally OD was recorded at 520nm using an ELISA plate reader. Urea concentration was calculated according to the manufacturer’s formula.

### Mouse histopathology staining

To avoid contamination of red blood cells and lymphocytes in kidney blood vessels, perfusion was performed pumping 10 ml sterile PBS from the left ventricle, while the right atrium was opened with a small cut. Mouse kidneys were fixed in 10% neutral buffered formalin and embedded in paraffin for histological and immunohistochemical analyses. Thereafter, specimens were dehydrated in an ascending ethanol series and embedded in paraffin for subsequent sectioning into 5–7 μm sections using a microtome (Leica). Three or four consecutive renal tissue sections were placed onto a slide for histological analyses. Mouse renal tissue sections were stained with H&E or Jones’ methenamine silver-periodic acid-Schiff according to standard practices.

One observer blinded to the experimental design evaluated histopathology. The severity of GN was scored as previously reported [5]. GN severity results are presented as the sum of proliferative and chronic glomerular pathology.

### B cell purification

Splenic B cells were purified by negative selection using magnetic beads and MACS separation columns. Briefly, spleen cells from littermate mice were labeled with a cocktail of biotin-conjugated antibodies for 15 minutes. Cells were incubated an additional 15 minutes with anti-biotin microbeads (B Cell isolation Kit, mouse; Miltenyi) at 4°C. The labeled non-B-cells were depleted by magnetic retention in a MACS column while unlabeled B cells were recovered. The purity of the resulting cell population was typically more than 96% either B220^+^CD3^-^ or CD19^+^CD3^-^ as assessed by flow cytometry analysis.

### Quantitative RT-PCR

Total RNA was isolated by Trizol method (Invitrogen) from 1×10^6^ splenic B cells. After Nanodrop, 500ng RNA was subjected to first-strand cDNA synthesis for qRT-PCR (Origene). Five nanograms of total cDNA/RNA were used per reaction with TaqMan Fast Universal PCR Master Mix. The qRT-PCR reactions were performed on a 7900 HT Fast RealTime instrument. The primers and probes for mouse *Aicda* (Assay ID: Mm00507777_g1 and Mm01184115_m1), *Ifnb1* (Assay ID: Mm00439552_s1), *Irf1* (Assay ID: Mm01288580_m1), *Irf5* (Assay ID: Mm00496477_m1), *Irf7* (Assay ID: Mm00516793_g1), *Irf9* (Assay ID: Mm00492679_m1), *Stat1* (Assay ID: Mm00439531_m1), *Stat3* (Assay ID: Mm01219775_m1) and *Tlr7* (Assay ID: Mm00446590_m1) were purchased from Taqman Gene expression Assays (Life Technologies).

### Western Blotting and Nuclear Extract Preparation

Stimulated and unstimulated splenic B cells were lysed in buffer containing 1% TritonX-100, 50 mM Tris pH 7.4, 50 mM NaCl, 1 mM EDTA, 2 mM Na_3_VO_4_, and a protease inhibitor cocktail (Roche Applied Science) to be prepared as whole cell lysates except for subcellular fractionation experiments. LDS sample buffer and reducing agent (Life Technologies) were added. Samples were then boiled, and protein was separated with NuPAGE 4–12% Bis-Tris Gels (Life Technologies). Proteins were blotted onto polyvinylidene difluoride membranes, and the immunoblots were processed with specific antibodies. Nuclear extracts, cytosolic extracts, and whole cell lysates were prepared as per the manufacturer’s instructions by using “Nuclear Extract Kit” (Active Motif) for IRF7 nuclear translocation experiments.

All phospho-specific antibodies and phosphorylation-state-independent antibodies for p38, Jnk, Erk, IκBα, Stat1, Irf7; and for cytosol specific Rho A and nuclear specific Lamin B1 and Gapdh loading control were all purchased from Cell Signaling Technology. Detection of activated and total Mnk1/2 and eIF4E was done as previously described [5].

### Statistics

Statistical significance between 2 data sets was determined by Mann-Whitney nonparametric test. When necessary, two-way analysis of variance (ANOVA) or one- way ANOVA was performed. For all tests, *P*<0.05 was considered statistically significant. Graphs and statistical analyses were performed using Prism 6.0 software (GraphPad). Values are reported as mean with or without SEM and/or SD.

## Results

### *Bank1* Deficiency reduces total IgG and IgG anti-dsDNA antibodies in B6.Sle1.yaa lupus mice

**Fig 1:**
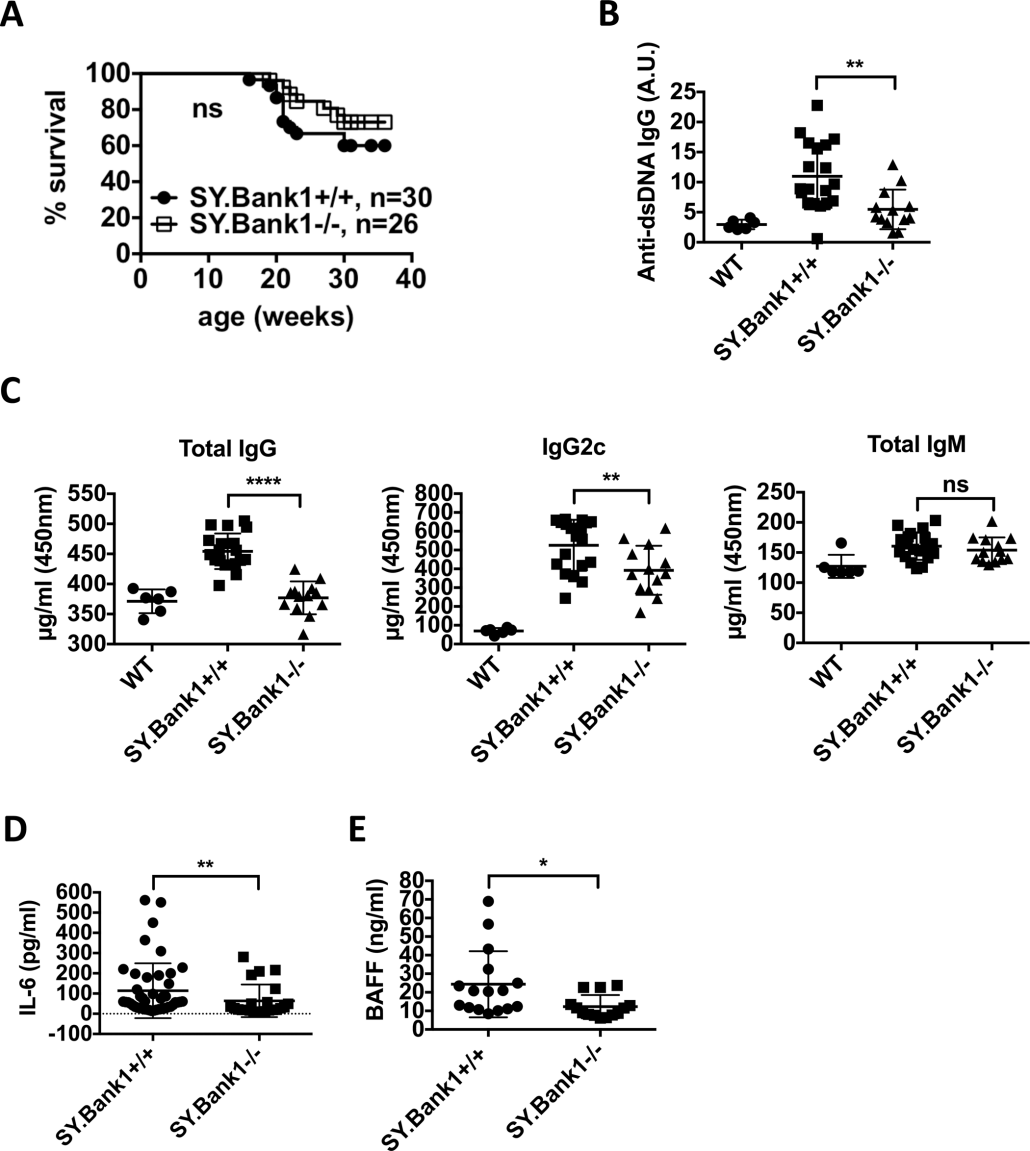
Effect of *Bank1* deficiency on major lupus phenotypes: *Bank1* deficiency reduces mortality and production of total IgG and IgG anti-dsDNA antibodies as well as levels of IL-6 and BAFF in serum. (A) Survival was monitored in two groups of B6.*Sle1*.*yaa*.*Bank1*^*+/+*^ (*SY*.*Bank1*^*+/+*^; n = 30) and B6.*Sle1*.*yaa*.*Bank1*^*-/-*^ (*SY*.*Bank1*^*-/-*^; n = 26) mice up to 36 weeks. A Kaplan-Meier survival plot is shown. The Gehan-Breslow-Wilcoxon test was applied with a p value = 0.2032. (B) Serum titers of IgG anti-dsDNA antibodies from 20-24-week old mice. Sera were diluted 1:50. The experiment was in triplicates. Each point represents one mouse; Levels are expressed as arbitrary units (A.U.) (mean± SEM; **p≤0.001, nonparametric *t* test). Total mice analyzed: WT (n = 6), *SY*.*Bank1*^*+/+*^ (n = 19), *SY*.*Bank1*^*-/-*^ (n = 13). (C) Serum total IgG, IgG2c and total IgM quantified by ELISA from 20~24 wk-old mice. For IgM and IgG sera were diluted 1:200, whereas for IgG2c 1:10000. Experiments were in duplicates. Each point represents one mouse; bars show mean ±SD. The levels of significance were compared between *SY*.*Bank1*^*+/+*^ and *SY*.*Bank1*^*-/-*^ mice by t-test, **p = 0.0098, ****p<0.0001 and n.s. not significant. Total mice analyzed: *SY*.*Bank1*^*+/+*^ (n = 19), *SY*.*Bank1*^*-/-*^ (n = 13). (D) Serum IL-6 from 18-wks mice diluted 1:10. Total mice analyzed: *SY*.*Bank1*^*+/+*^ (n = 46), *SY*.*Bank1*^*-/-*^ (n = 23). Bars show mean ± SD. **p = 0.0067. (E) BAFF levels in 18 weeks sera from *SY*.*Bank1*^*+/+*^ (n = 16) and *SY*.*Bank1*^*-/-*^ (n = 14) mice. Sera were diluted 1:24 and experiments done in duplicates. Each point represents the value from one mouse; bars show mean ± SD; t-test was applied to get level of significance. *p = 0.0151.

To determine if deficiency of *Bank1* had any effects on disease phenotypes and disease progression, we began by analyzing the effects of *Bank1* deficiency on major lupus phenotypes. *Bank1* deficiency increased the survival rate of *Sle1*.*yaa* mice from 63% to 83% by week 24 and from 57% to 68% by week 36 (Fig 1A). The difference was, however, not significant.

In serum, *Bank1* deficiency in the B6.*Sle1*.*yaa* background was able to reduce IgG anti-dsDNA autoantibodies significantly by 24 weeks of age to levels comparable to those of wild-type mice (Fig 1B). Most significant was the reduction of total IgG antibodies in serum followed by total IgG2c in B6.*Sle1*.*yaa*.*Bank1*^*-/-*^ mice as compared to B6.*Sle1*.*yaa*.*Bank1*^*+/+*^ mice by 20–24 weeks of age. Total IgM level in the serum was not affected by *Bank1* deficiency (Fig 1C).

Interestingly, deficiency of *Bank1* had no effect on the development of the cumulative glomerulonephritis score (S1A Fig), no difference in levels of blood urea nitrogen (BUN) (S1B Fig) or histological nephritis in general (S1C Fig).

It has been previously shown that an *Il-6* knock-out mouse completely abrogates lupus phenotypes in B6.*Sle1*.*yaa* mice [28]. Consequently we measured IL-6 in the serum of the animals. Levels of serum IL-6 were decreased in the B6.*Sle1*.*yaa*.*Bank1*^*-/-*^ mice by week 18 as compared to B6.*Sle1*.*yaa*.*Bank1*^*+/+*^ mice (Fig 1D) but the difference was no longer significant by week 24 (S1D Fig). This was probably due to the death of several B6.*Sle1*.*yaa*.*Bank1*^*+/+*^ mice at 18- and 24-weeks of age.

The B cell activating factor belonging to the TNF family (BAFF) is a survival and differentiation factor for B cells and an important target for therapy in SLE [29], as it has been shown that overexpression of BAFF augments the development of SLE [30]. We analyzed levels of BAFF in the serum of our mice. Our results show that BAFF was modestly, but significantly reduced in B6.*Sle1*.*yaa*.*Bank1*^*-/-*^ mice by week 18 (Fig 1E).

### *Bank1* deficiency restores to normal levels of spleen and bone marrow B cell subsets, and expression of CXCR4 on follicular T helper cells

**Fig 2:**
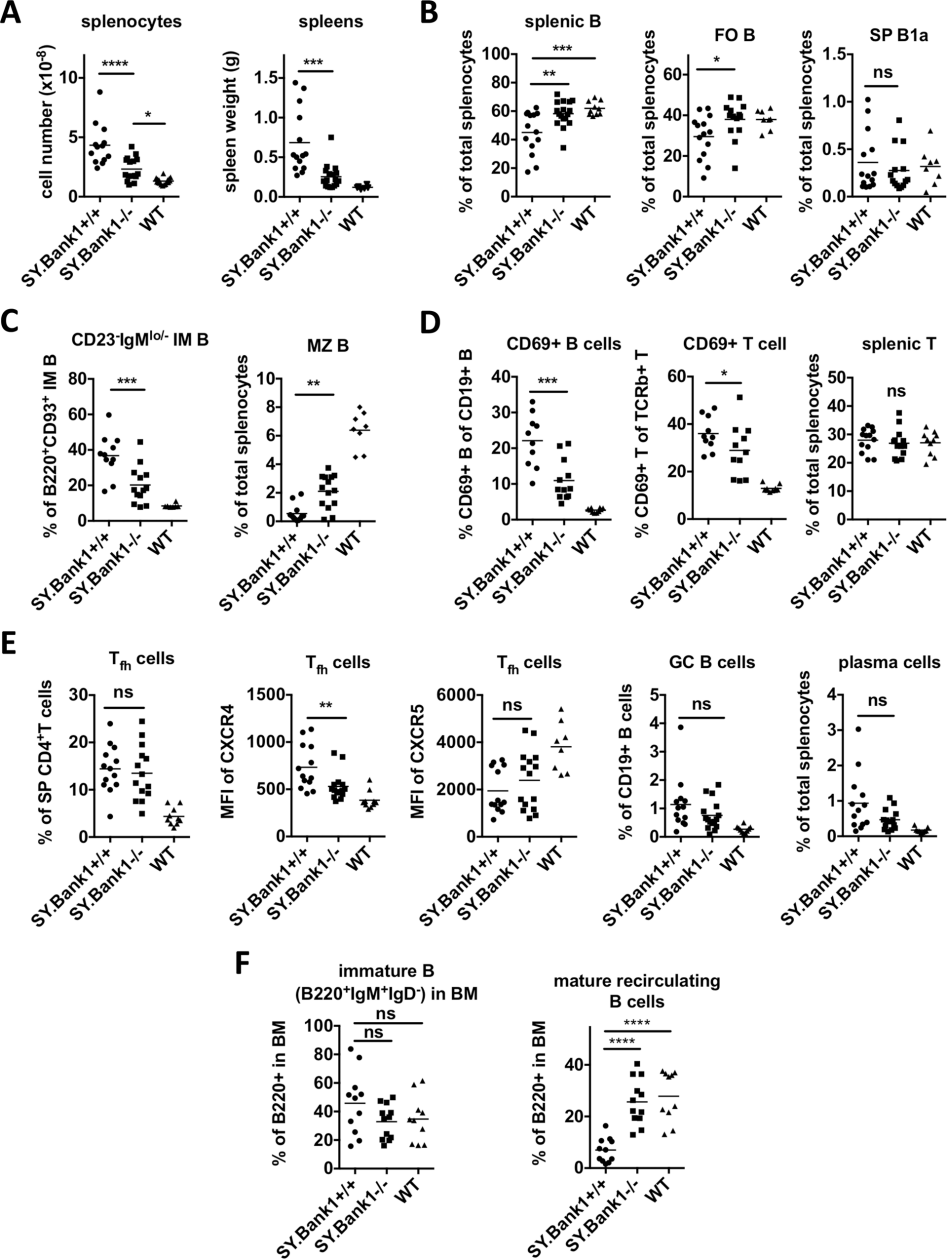
*Bank1* deficiency restores the cellular phenotypes of splenic lymphocytes from B6.*Sle1*.*yaa*.*Bank1*^*+/+*^ mice. (A) Splenocyte numbers (left) and spleen weight (right) in *SY*.*Bank1*^*-/-*^ and *SY*.*Bank1*^*+/+*^ mice. (B) *S*plenic B cells (left), follicular B cells (FO B; middle) and splenic B1a subset in *SY*.*Bank1*^*+/+*^ and *SY*.*Bank1*^*-/-*^ mice. Total mice analyzed: *SY*.*Bank1*^*+/+*^ (n = 14), *SY*.*Bank1*^*+/+*^ (n = 15), WT (n = 8). (C) CD23^-^IgM^lo/-^ immature B cells in the spleen characterized as B220^+^CD93^+^CD23^-^IgM^lo/-^. The data are shown as percentage of B220^+^CD93^+^ immature B cells (IM B; left). Marginal zone B (MZ B) cells are characterized as B220^+^CD93^-^CD23^lo^CD21^hi^. Total mice analyzed: *SY*.*Bank1*^*+/+*^ (n = 11), *SY*.*Bank1*^*-/-*^ (n = 12 for immature B cells, n = 14 for MZ B), WT (n = 8). (D) Activation status of CD19^+^ splenic B cells and splenic T cells as determined by percentage of CD69^+^ cells out of CD19^+^B cells or TCRβ^+^T cells, and the frequency of splenic T cells in *SY*.*Bank1*^*+/+*^, *SY*.*Bank1*^*-/-*^ and WT mice. Total mice analyzed: *SY*.*Bank1*^*+/+*^ (n = 10; n = 11 for splenic T), *SY*.*Bank1*^*-/-*^ (n = 11; n = 12 for splenic T), WT (n = 10). (E) The frequency of follicular helper T (T_fh_) cells in splenic CD4^+^T and expression levels (MFI) of CXCR4 and CXCR5 in *SY*.*Bank1*^*-/-*^ and *SY*.*Bank1*^*+/+*^mice, and germinal center B (GC B; CD19^+^IgD^-^PNA^hi^GL7^+^) cells and plasma cells (CD11c^-^CD3^-^B220^int/-^CD138^+^) populations. (F) Immature B cells (B220^+^IgM^+^IgD^-^) and mature recirculating B cells (B220^+^IgM^+^IgD^hi^) from bone marrows. Total mice analyzed: *SY*.*Bank1*^*+/+*^ (n = 11), *SY*.*Bank1*^*-/-*^ (n = 11), WT (n = 10). Data pooled from 4 independent experimental cohorts of mice. Statistical plots are shown as mean with Mann-Whiney (*SY*.*Bank1*^*+/+*^ vs. *SY*.*Bank1*^*-/-*^) nonparametric test (*p≤0.05, **p≤0.01, ***p≤0.001, ****p≤0.0001, ns>0.05).

BANK1 is primarily expressed in B cells [2]. We investigated whether *Bank1* deficiency could restore to normal the cellular phenotypes modified by the autoimmune process in B6.*Sle1*.*yaa* splenic B cells. B6.*Sle1*.*yaa*.*Bank1*^*+/+*^ mice showed the expected splenomegaly, increased weight and increased spleen cell numbers, whereas *Bank1* deficiency reduced the splenomegaly to a significant extent, without reaching that of normal B6 mice (Fig 2A). On the other hand, frequencies of splenic B cells and the main B cell subset, follicular B (FO B) cells, abnormally reduced in B6.*Sle1*.*yaa*.*Bank1*^*+/+*^ mice, were restored to the levels of normal B6 mice by the *Bank1* deficiency (Fig 2B, and gating strategy in S2A Fig).

When analyzing mature B cell subpopulations, we confirmed that MZ B cell development was impaired in B6.*Sle1*.*yaa*.*Bank1*^*+/+*^ mice [24]. *Bank1* deficiency partially restored MZ B cell frequency (Fig 2C, gating strategy in S2A Fig) while the proportion of splenic B1a cells was not changed (Fig 2B, gating strategy in S2A Fig). We also characterized immature B cell populations, particularly transitional 1, 2, and 3 B cell subsets. Frequencies of T1, T2, and T3 B cell subsets were not changed by *Bank1* deficiency (S2B Fig). Interestingly, we found that the CD23^-^IgM^lo/-^ immature B cell population, importantly increased in B6.*Sle1*.*yaa* mice, was significantly reduced in the *Bank1* deficient mice (Fig 2C and S2B Fig; gating strategy in S2A Fig).

Spontaneous B cell activation is a hallmark of SLE. As expected, B cell activation, as measured by surface CD69 expression, was increased in B6.*Sle1*.*yaa*.*Bank1*^*+/+*^ mice and was significantly reduced (Fig 2D, gating strategy in S2C Fig) in B6.*Sle1*.*yaa*.*Bank1*^*-/-*^ mice.

Overall, *Bank1* deficiency did not affect splenic T cell frequencies of B6.*Sle1*.*yaa*.*Bank1*^*+/+*^ mice (Fig 2D), however, there was a weak, but significant suppression of T cell activation by downregulation of CD69 expression on T cells (Fig 2D, gating strategy in S2C Fig).

Both adaptive and innate immunity are induced and show abnormalities in B6.*Sle1*.*yaa* mice [26]. To analyze the effects of *Bank1* deficiency on phenotypes associated with adaptive immune responses, we focused on follicular helper T (T_fh_) cells, germinal center (GC) B cells and plasma cells. Splenic T_fh_ cell frequency was not different between B6.*Sle1*.*yaa*.*Bank1*^*+/+*^ mice and B6.*Sle1*.*yaa*.*Bank1*^*-/-*^ mice (Fig 2E, gating strategy in S3A Fig based on [31]). However, *Bank1* deficiency downregulated surface expression of CXCR4 on T_fh_ cells, while expression of CXCR5 was not modified (Fig 2E, S3A and S3B Fig). Germinal center B cell frequency of B6.*Sle1*.*yaa*.*Bank1*^*-/-*^ mice had a non-significant tendency towards levels comparable to those of C57BL/6 mice (Fig 2E and S3C Fig). The presence of CD138^+^ plasma cells from B6.*Sle1*.*yaa*.*Bank1*^*-/-*^ mice was slightly reduced but not significantly (Fig 2E, gating strategy in S3D Fig).

Next we investigated whether *Bank1* deficiency could restore to normal the cellular populations of the bone marrow (BM) altered in B6.*Sle1*.*yaa* mice. *Bank1* is not expressed in B cell precursors (eg. pro-B, and pre-B cells) until B cell development proceeds to the immature B cell stage in the bone marrow [2], so we checked immature B cells (B220^+^IgM^+^IgD^-^), as well as re-circulating mature B cells (B220^+^IgM^+^IgD^hi^) [32] (S4 Fig). The frequency of immature B cells in BM was not different between the two mouse genotypes. In contrast, the percentage of re-circulating mature B220^+^ B cells in the BM of B6.*Sle1*.*yaa*.*Bank1*^*+/+*^ was <10% on average, while that of B6.*Sle1*.*yaa*.*Bank1*^*-/-*^ reached on average 26% of BM cells, frequencies comparable to those of WT mice BM (Fig 2F).

### *Bank1* deficiency corrects splenic myeloid cell numbers of B6.*Sle1*.*yaa* mice

**Fig 3:**
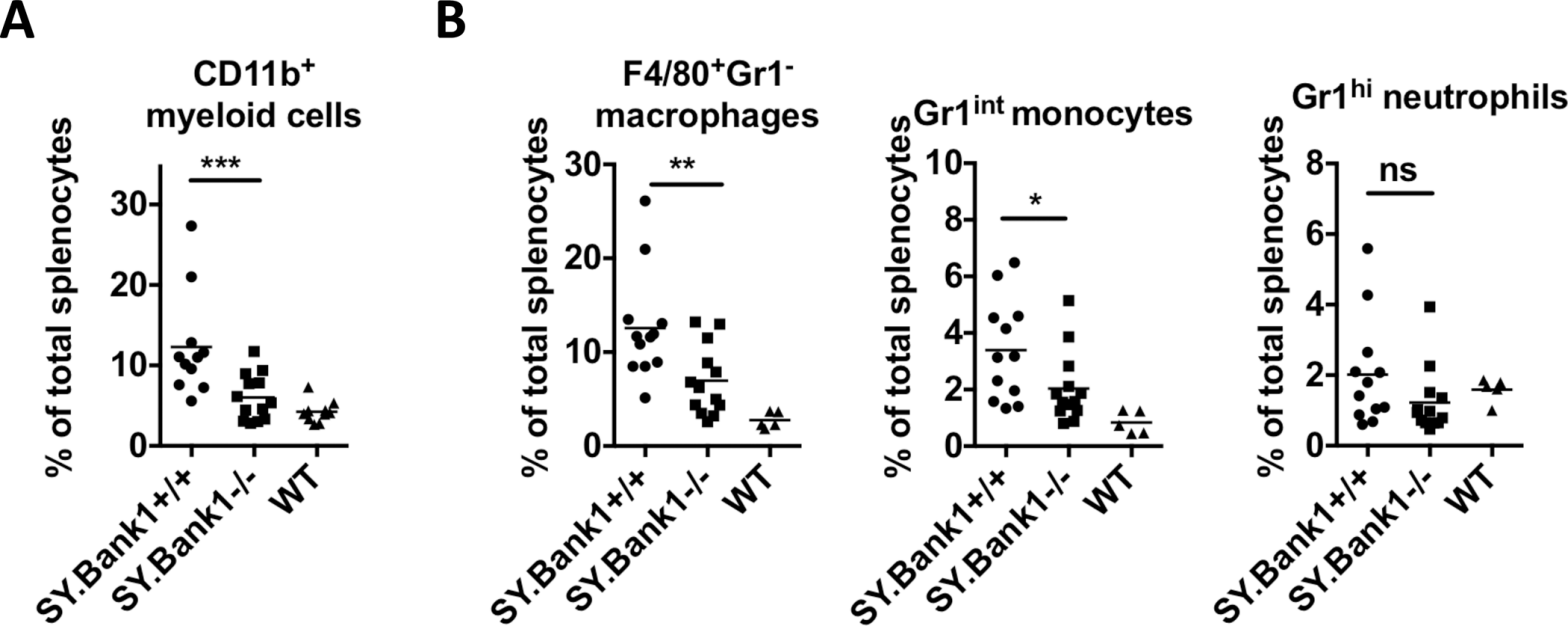
*Bank1* deficiency restores to normal the frequencies of spleen myeloid cells. (A) Statistical plots frequencies of CD11b^+^ myeloid cells in total splenoctyes of *SY*.*Bank1*^*-/-*^, *SY*.*Bank1*^*+/+*^ and WT mice. (B) Frequencies of splenic F4/80^+^Gr1^-^ macrophages, Gr1^int^ monocytes, and Gr1^hi^ neutrophils. Total mice analyzed: *SY*.*Bank1*^*+/+*^ (n = 12), *SY*.*Bank1*^*-/-*^ (n = 13), WT (n = 5). Data pooled from 4 independent experimental cohorts of mice. Statistical plots are shown as mean with Mann-Whitney (*SY*.*Bank1*^*+/+*^ *vs*. *SY*.*Bank1*^*-/-*^) nonparametric test (*p≤0.05, **p≤0.01, ***p≤0.001, ns>0.05).

Chronic inflammation in B6.*Sle1*.*yaa* mice leads to the skewing of hematopoiesis towards the myeloid lineage [33]. We found that *Bank1* deficiency could almost completely normalize hematopoietic cell development leading to a restoration of CD11b^+^ myeloid cells in spleens (Fig 3A, gating strategy in S5A Fig), as well as F4/80^+^ macrophages, and Gr1^int^ monocytes (Fig 3B, gating strategy in S5B Fig). On the other hand, *Bank1* deficiency did not affect the development of Gr1^hi^ neutrophils (Fig 3B and S5B Fig).

### *Bank1* deficiency impaired the expression of TLR7 signaling-dependent genes and STAT1 activation

**Fig 4:**
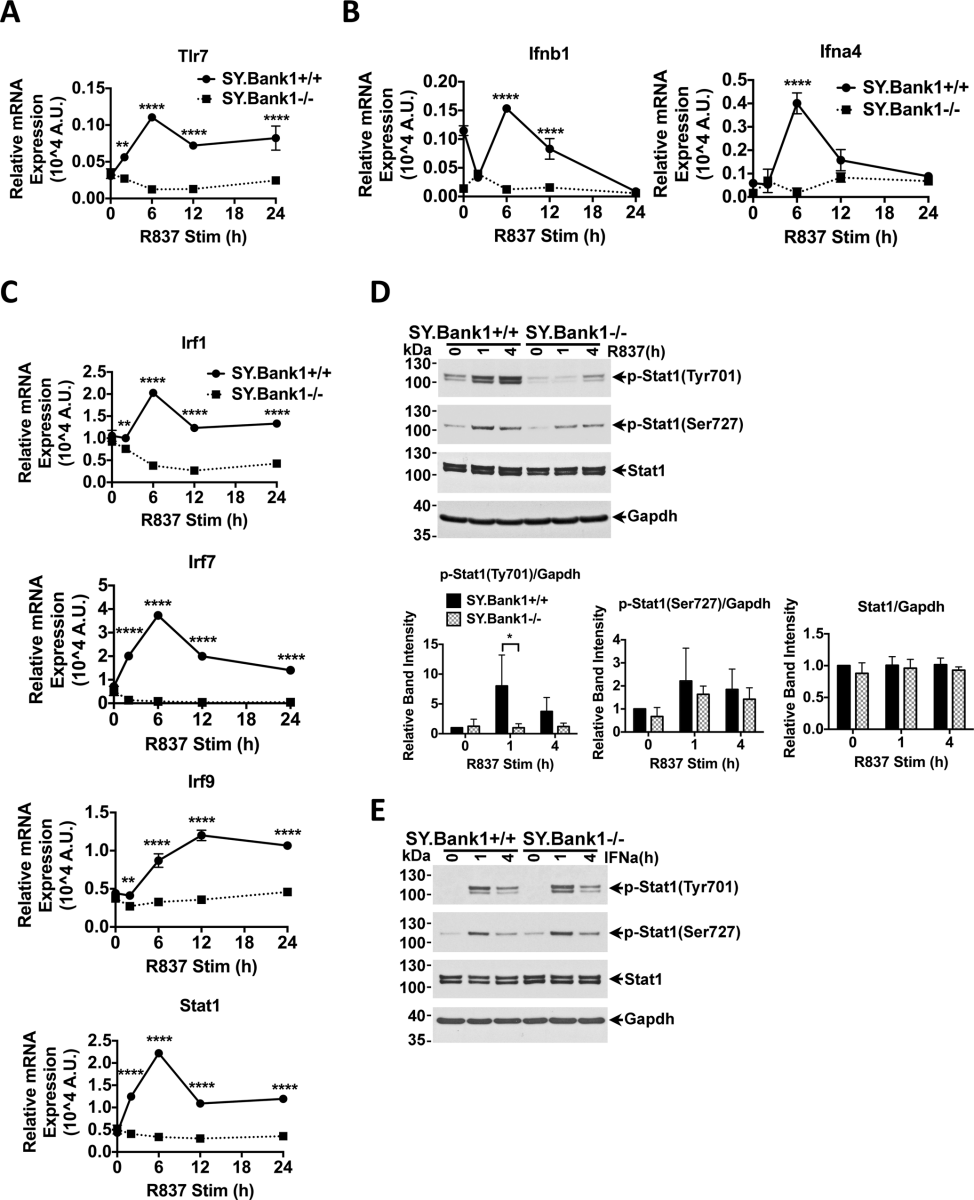
Effects of *Bank1* deficiency on the *in vitro* expression of TLR7-induced genes from purified splenic B cells of B6.*Sle1*.*yaa* mice. Quantitative RT-PCR analysis of genes in splenic B cells stimulated with TLR7 agonist (R837, 2μg/ml) for the indicated periods. Expression was normalized to 18s rRNA control gene. Results are representative of two-independent experiments. The genes that are shown are: (A) *Tlr7*. (B) *Ifnb and Ifna4*. (C) *Irf1*, *Irf7*, *Irf9* and *Stat1* gene expression. Purified B cells from *SY*.*Bank1*^*+/+*^ and *SY*.*Bank1*^*-/-*^ mice were stimulated with (D) R837 (2 μg/ml) or (E) rIFNα (2000 U/ml) for the indicated periods and analyzed by immunoblotting with phospho-Stat1 (Tyr701), phospho-Stat1 (Ser727) and total Stat1 antibodies. Gapdh was used as control. Blots are representative of 3 independent experiments. Bar plots represent the mean ±SD of the relative band intensity of phospho-Tyr701 and–Ser727 of Stat1 and total Stat1 proteins obtained from three independent experiments. Mann-Whitney nonparametric test (*SY*.*Bank1*^*+/+*^ *vs*. *SY*.*Bank1*^*-/-*^) (*p≤0.05, **p≤0.01, ****p≤0.0001, ns>0.05, not shown).

We then investigated if *Bank1* deficiency could affect the expression of genes directly induced by TLR7 stimulation in the B6.*Sle1*.*yaa* mice. We therefore stimulated purified splenic B cells from B6.*Sle1*.*yaa*.*Bank1*^*+/+*^ and B6.*Sle1*.*yaa*.*Bank1*^*-/-*^ mice with the TLR7 agonist R837 (Imiquimod). First, our results showed that *Tlr7* expression at baseline was not altered in *Bank1*-deficient splenic B cells (Fig 4A), but expression was subsequently induced in the B6.*Sle1*.*yaa*.*Bank1*^*+/+*^ splenic B cells following stimulation. Thus, the data showed a significant difference in *Tlr7* gene expression between the two genotypes and suggested that the impaired induction of TLR7-mediated type I IFN production or signaling caused by the *Bank1* deficiency may lead to the maintenance of the basal levels of *Tlr7*. To this end, we examined expression of *Ifnb and Ifna4* following TLR7 agonist stimulation. Expression of *Ifnb and Ifna4* by splenic B cells was clearly not induced in B6.*Sle1*.*yaa*.*Bank1*^*-/-*^ mice, but largely induced in B6.*Sle1*.*yaa*.*Bank1*^*+/+*^ mice (Fig 4B). We then investigated the expression of several interferon regulatory factors (*Irfs*), *Stat1* and *Stat3* under the same conditions. We observed the lack of induction of the basal levels of expression of the genes *Irf1*, *Irf7*, *Irf9*, and *Stat1* in B6.*Sle1*.*yaa*.*Bank1*^*-/-*^ mice, but induction was present in B6.*Sle1*.*yaa*.*Bank1*^*+/+*^ mice (Fig 4C). In contrast, *Irf5* and *Stat3* gene expression did not show significant difference upon TLR7 agonist stimulation between B6.*Sle1*.*yaa*.*Bank1*^*-/-*^ mice and B6.*Sle1*.*yaa*.*Bank1*^*+/+*^ mice (S6A Fig).

STAT1 becomes activated either through TLR-mediated MAPK p38 phosphorylation (phosphorylation of Ser727) or through the type I IFN receptor (phosphorylation of Tyr701) [34]. IFN can be taken up from external sources by B cells, for instance that produced by pDCs, or by the autocrine loop, where B cell IFN production is enhanced together with cytokine and antibody secretion [17]. Therefore, we determined the effects of *Bank1* deficiency on STAT1 activation following imiquimod stimulation (Fig 4D). Levels of phospo-Tyr701 activated STAT1 were already reduced in the B6.*Sle1*.*yaa*.*Bank1*^*-/-*^ unstimulated B cells, and after stimulation activation remained extremely low as compared to purified splenic B cells from the B6.*Sle1*.*yaa*.*Bank1*^*+/+*^ mice (Fig 4D). Activation of Ser727 STAT1 was not modified (Fig 4D), suggesting that *Bank1* deficiency is not acting through TLR7-induced p38 activation, but rather through the JAK/STAT pathway possibly as a consequence of the already reduced expression of type I IFN following TLR7 stimulation.

To determine if the effect of *Bank1* deficiency was on the TLR7 pathway and not the result of the feedback loop of type I interferon through the IFN receptor, we investigated the expression of the *Irf* genes, *Stat1* and *Stat3*, as well as STAT1 activation following stimulation with recombinant IFNα (rIFNα). None of the *Irf* or *Stat* genes studied was modified when exogenous rIFNα was added to the cell culture (S6B Fig). Importantly, STAT1 activation was not modified by the absence of *Bank1* following rIFNα stimulation (Fig 4E).

### *Bank1* deficiency impaired the IL-6 and IgG2c production in response to TLR7 agonist stimulation *in vitro*

**Fig 5:**
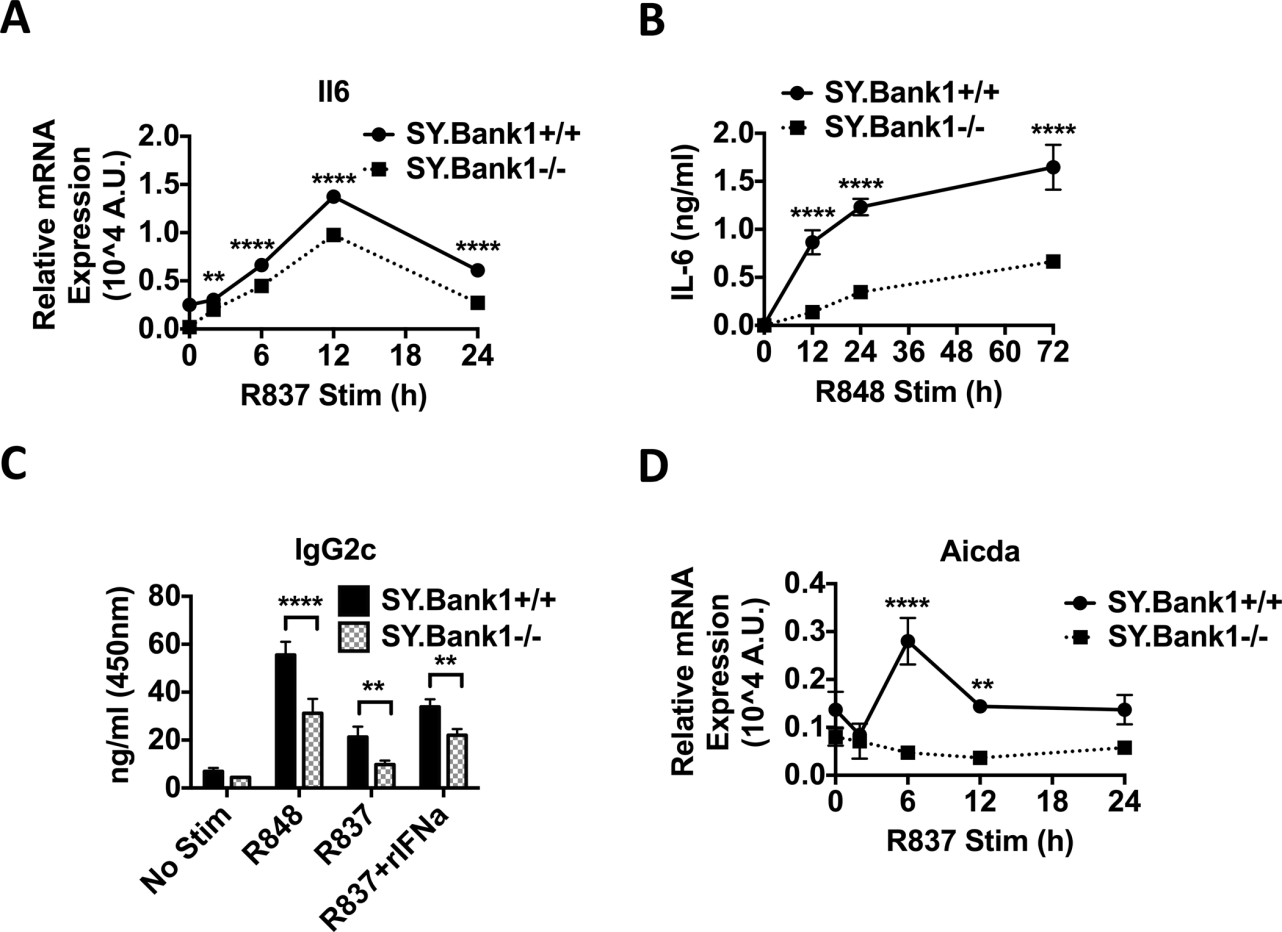
Reduced IL-6 and IgG2c antibody production in *Bank1*-deficient mice after stimulation with TLR7 agonists. (A) Quantitative RT-PCR analysis of *Il6* gene expression. Experiment was performed as in (Fig 4A). (B) IL-6 cytokine secretion in supernatants from purified B cells stimulated with R848 (1 μg/ml, another TLR7 agonist) in triplicates for the time points shown. (C) IgG2c production in supernatants of purified splenic B cells stimulated with R837, R848, and rIFNα + R837, for various time points is reduced in *Bank1*-deficient mice. Bars represent the average of triplicates ±SD. Two-way ANOVA with Sidak’s multiple comparisons test was applied. ****p<0.0001. Data is representative 3 independent experiments. (D) Expression of the activation-induced cytidine deaminase gene *Aicda*. Experiment was performed as in Fig 4A.

Serum IL-6 levels were reduced in B6.*Sle1*.*yaa*.*Bank1*^*-/-*^ mice (Fig 1E) and we have previously shown that *Bank1* deficiency reduces IL-6 production by altering the translation initiation pathway in a p38-dependent manner and following TLR9 agonist stimulation [5]. Thus we investigated the expression of the pro-inflammatory cytokine *Il6* gene after stimulation of splenic B cells with TLR7 agonists. Expression of the *Il6* gene was slightly reduced (Fig 5A), however secretion of IL-6 protein was importantly reduced after TLR7 stimulation and remained reduced throughout the 3 days of *in vitro* culture (Fig 5B).

TLR7 stimulation induces not only the secretion of type I interferon and pro-inflammatory cytokines such as IL-6, but also the production of antibodies. IgG2a antibodies, correspondingly IgG2c in C57BL/6 mice, are increased in autoimmune models of lupus [19, 35]. Production of IgG2c antibodies in the supernatants was importantly reduced after 6 days of TLR7 stimulation in B6.*Sle1*.*yaa*.*Bank1*^*-/-*^ mice as compared to the B6.*Sle1*.*yaa*.*Bank1*^*+/+*^ mice (Fig 5C). We then investigated if absence of *Bank1* could affect expression of the activation-induced cytidine deaminase (*Aicda*) gene involved in IgG class switching. Consistent with reduction of total IgG observed in serum (Fig 1C) and the reduction of IgG2c in culture supernatants, the expression levels of *Aicda* gene were reduced upon TLR7 agonist stimulation, but not rIFNα stimulation in the *Bank1*-deficient B6.*Sle1*.*yaa* mice (Fig 5D and S6C Fig).

### Bank1 does not alter MAPK or NFκB signaling pathways following TLR7 agonist stimulation

We had previously shown that *Bank1* deficiency led to a reduction in CpG-induced p38 signaling specifically affecting the activation of MNK1/2 and eIF4E [5]. To determine if we could observe a similar effect under TLR7 stimulatory conditions in the B6.*Sle1*.*yaa*.*Bank1*^*-/-*^ mice, we stimulated purified splenic B cells with R848 followed by analysis of MAP kinase and NFκB activation by Western blot (S7A Fig). We observed no effects on JNK or ERK activation at any of the time points measured and no changes in p38 and NFκB activation measured by phosphorylation of IκB and IκB degradation (S7B Fig). The observed results support the notion that *Bank1* does not affect TLR7 signaling through p38 MAPK.

### *Bank1* deficiency reduces IRF7 expression and its nuclear translocation upon TLR7 stimulation

**Fig 6:**
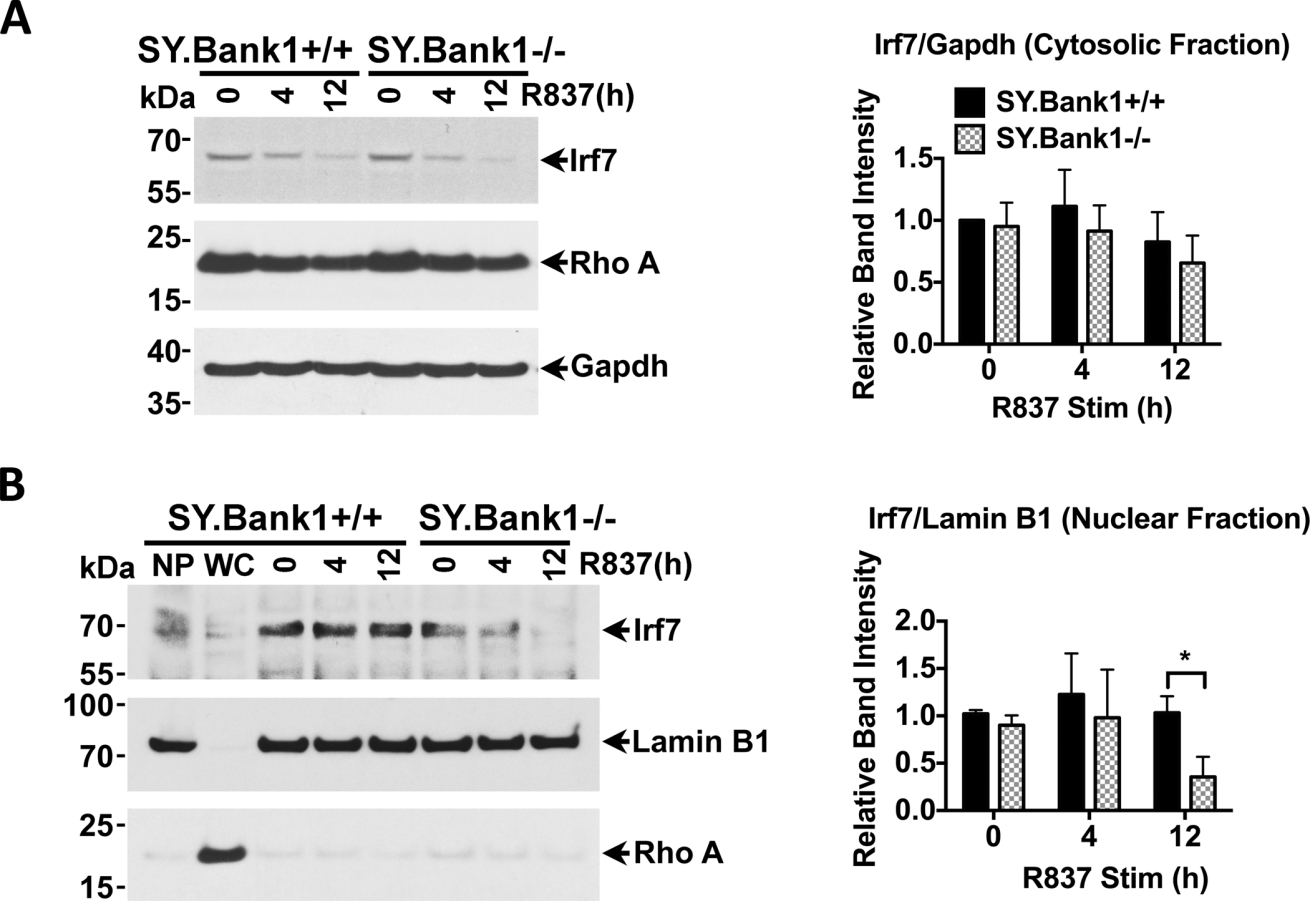
*Bank1* deficiency reduces the nuclear translocation of IRF7. Levels of IRF7 protein in the (A) cytosol and the (B) nucleus following TLR7 agonist stimulation for the indicated times was detected by immunoblot on purified splenic B cells. Gapdh and Lamin B1 were used as control for the cytosolic and nuclear fractions, respectively. Statistical column plots present the relative band intensity relative to Gapdh (A) and Lamin B1 (B) of cytosolic and nuclear Irf7, respectively. NP: nuclear pellet; WC: whole cell lysate. Statistical plots are shown as mean with SD of 3 independent experiments. Mann-Whitney nonparametric test (*SY*.*Bank1*^*+/+*^ *vs*. *SY*.*Bank1*^*-/-*^) (*p≤0.05, ns>0.05 [not shown]).

One of the major effectors of TLR7 signaling is IRF7 on which *Ifnb* expression is dependent. As we observed reduction in *Ifnb* and *Irf7* expression, we verified if we observed reductions in IRF7 protein. Total IRF7 protein is distributed in both the cytoplasm and the nucleus. Prior to stimulation, IRF7 levels in the cytosol-enriched and nuclear fractions were similar in the purified B cells from both mouse strains (Fig 6A and 6B). On the other hand, nuclear IRF7 was significantly reduced in the *Bank1*-deficient B cells as compared to the *Bank1*-sufficient B cells at 12 hr after R837 stimulation (Fig 6B). Thus, Bank1 is involved in the nuclear translocation of IRF7.

### The role of Bank1 in the Mnk1/2-eIF4E-mediated translation initiation pathway induced by type I IFN

Type I IFN is essential for the downstream effects of TLR7 stimulation on B cell function. In fact, the optimal secretion of cytokines induced by TLR7 stimulation can only be attained through the positive feedback loop requiring the production of IFN that is then introduced into the cell through the IFNAR, stimulating the JAK/STAT pathway. We have shown that *Bank1* deficiency did not modify the activity of STAT1 when stimulated by rIFNα (Fig 4D and 4E) and observed no changes in p38 activity following TLR7 stimulation (S7A Fig). It has been shown that IFNα can induce the eIF4E translation initiation pathway [36] and that this pathway mediates the translation of interferon-inducible genes. The type I IFN signature is key in the development of SLE, and its modification may depend, at least partly on 5´cap protein translation [36, 37]. The type I IFN induced translation initiation activates Mnk1/2 and eIF4E, independently of p38 [36]. We verified the activation of these proteins in the B6.*Sle1*.*yaa*.*Bank1*^*+/+*^ and the B6.*Sle1*.*yaa*.*Bank1*^*-/-*^ mice. Our results show that with rIFNα alone the activation of p38 was not significantly reduced at any time point after stimulation in the *Bank1* deficient mice (S8A Fig). On the other hand, only phosphorylation of Mnk1/2, but not phosphorylation of eIF4E, was significantly reduced in purified splenic B cells from B6.*Sle1*.*yaa*.*Bank1*^*-/-*^ mice (S8B and S8C Fig). Our data does not support an effect of the *Bank1* deficiency on the activation of the translation initiation complex induced by type I IFN.

## Discussion

Our results suggest that deficiency of *Bank1* limits lupus endo-phenotypes by reducing the production of type I IFN by B cells, disturbed B lymphoid and myeloid lineage development, the production of IgG antibodies, and the production of pro-inflammatory cytokines such as IL-6, all constantly under enhanced pressure by the excessive expression of *Tlr7* found in the *Yaa* locus.

Though the role of Bank1 on the development of glomerulonephritis and mortality is not demonstrated, the results are consistent with the findings of Hwang et al. [38] where not only B cells, but also myeloid cells, are involved in lupus pathogenesis. Because *Bank1* is primarily expressed in B cells, our results imply that impairment of TLR7 signaling in B cells is not sufficient to trigger autoimmunity. It should also be pointed out that SLE in mice and humans is a complex disease that requires the sum and interaction of multiple genes and that in general, the effects of these genes are subtle on an individual basis. Our results therefore show a significant impact of *Bank1* deficiency at the cellular level and on TLR7 signaling, but those phenotypes are not reflected on the disease clinical manifestations or on mortality.

It could be argued that as pDCs are the major producers of type I IFN, the IFN production by B cells is of minor importance. However, the absence of myeloid cell expansion and autoimmunity in *IgH*^*-/-*^ mice in the presence of overexpression of TLR7 shows that the innate immune response role of B cells in the disease is fundamental [15]. These data are in agreement with our observations of reduced myeloid cells in the B6.*Sle1*.*yaa* mice without *Bank1*. Furthermore, it has been shown that chronic inflammation and excessive and chronic type I IFN secretion can skew hematopoiesis towards the myeloid lineage [33].

A major striking difference was a reduction of total serum IgG but not IgM. Intriguingly such reduction of IgG was not accompanied by differences in numbers of germinal center B cells or plasma cells suggesting that the effect of *Bank1* is not on the differentiation towards GC or PCs, but at the level of class switch recombination (CSR) and IgG secretion. Deficiency of *Bank1* reduced the expression of the activation-induced cytidine deaminase (*Aicda*) gene, as well as interferon response factor genes (*Irf1*, *Irf7*, *Irf9)*, *Stat1 and Ifnb* induced by TLR7 agonist stimulation. Our results are supported by previous data showing that *Irf9* and *Stat1* control IgG autoantibody production during strong upregulation of TLR7 expression [39]. In addition, STAT1 is required for expression of T-bet which has been shown to regulate CSR to IgG2a after T cell-independent B-cell stimulations. In turn, the Ets-1 transcription factor is required for STAT1-mediated T-bet expression [40] directly decreasing IgG2a in cytokine-stimulated B cells. It is therefore possible that *Bank1* expression has effects on *Ets1* expression. The effects of *Bank1*^*-/-*^ could be also due to reduced expression of IFNAR or IL-21R following TLR7 stimulation. These receptors, upon ligation by IFN or IL-21, respectively, are known to activate the JAK-STAT pathway and promote CSR [41].

As expected the deficiency of *Bank1* revealed most of its effects in B cells, including reduced B cell activation through the measurement of CD69 on the surface. Since B cells can mediate T cell-dependent humoral immune responses, a slight reduction of CD69 was also observed in T cells. However, the sole phenotype we observed in T cells and potentially the result of B-T interactions was the reduction in CXCR4 expression on T_fh_ cells. CXCR4 (CD186), a G-protein coupled receptor of CXCL12, is upregulated in several cell subsets in various lupus models, among them the *B6*.*Sle1*.*yaa* [42]. In that work, the authors observed that expression of CXCR4 was most probably modulated by IL-6 but not by type I IFN or TLR7 stimulation, and a consequence of disease. However, work by Fairhurst and her colleagues using a *Tlr7Tg*, showed that *Tlr7* overexpression did increase the expression of CD69 and CXCR4 in B and T cells, and also the expression of ICOS, important in cell migration and activation [38]. CXCR4^hi^ T cells are considered extrafollicular T helper cells [43]. These cells are believed to provide help to extrafollicular plasmablast responses in autoimmune-prone mice [44], through a mechanism involving ICOS. The reduced CXCR4 expression induced by *Bank1* deficiency in the B6.*Sle1*.*yaa* mice suggests that *Bank1* may indirectly regulate the expression of CXCR4 in these T_fh_ cells. As we observed no reduction in T_fh_ cell numbers or expression levels of CXCR5, it is skewing towards the migration of T_fh_ cells into the extrafollicular compartments in B6.*Sle1*.*yaa*.*Bank1*^*+/+*^ mice that is reversed in the B6.*Sle1*.*yaa*.*Bank1*^*-/-*^ mice.

Many of the abnormalities found in the B6.*Sle1*.*yaa* have been shown to occur in the context of the *Tlr7* overexpression even if it is solely expressed in B cells [38]. Of the B cell subsets, MZ B cells were the ones primarily altered, a result similar to what we observe in the *Bank1* deficient B6.*Sle1*.*yaa*. However, we do not observe changes in plasma cell or GC cell numbers. We observed a tendency to a reduction in plasma cell numbers, and our results are therefore not necessarily incompatible with those previously showing the effects of *Tlr7Tg* in B cells [38], further supporting a role for *Bank1* in TLR7 signaling together with the data on the changes in B and T cell activation.

Reduced activation of p38 with R848 stimulation in the presence of *Yaa* was not consistently observed (S7A Fig). This is to be compared with our previous data where TLR9 stimulation showed a reduction in the secretion of IL-6 that was dependent on a downstream branch of p38. p38 activation triggered Mnk1/2-eIF4E-controled translation initiation of IL-6. Activation of p38 under TLR7 signaling unlikely depends on *Bank1*, alternatively, the *Bank1*-mediated p38 activation in the context of TLR7 overexpression may be importantly overshadowed. According to Joshi, et al., the effect of type I IFN on Mnk1/2 and eIF4E is independent of p38, but dependent on MEK/ERK and JAK1 [36]. This is potentially the case in our experiments, as the reduction that we observe in p38 is very modest and occurs late, while that of Mnk1/2, but not eIF4E is more marked and a tendency maintained throughout all the time points tested. Much still needs to be done to understand the regulation of translation initiation in B cells.

Thus, in summary BANK1 regulates TLR7-induced signaling pathways in B cells.

The next step is to find the proteins interacting with BANK1 downstream of TLR7. This may provide the detailed molecular mechanism of how BANK1-mediated signaling under TLR7 regulates transcription of specific genes, particularly *Ifnb*, *Stat1* and *Irf* genes, controls the IRF7 nuclear translocation, and class switch recombination.

## Conclusions

Our results demonstrate that BANK1 is involved in TLR7-mediated type I IFN production that such involvement is important in the development of B cell and myeloid cell phenotypes typical of SLE. We are providing a sensible explanation of the role of BANK1 in autoimmunity and support for BANK1 as a susceptibility gene for the disease. We further dissect that the intrinsic effects of BANK1 in B cells modify TLR7 signaling. The absence of *Bank1* led to the reduction of type I IFN (*Ifna4 and Ifnb1*), the expression of TLR7-dependent genes, and nuclear translocation of IRF7, as well as the reduced activation of STAT1. This reduced STAT1 activation was on the tyrosine 701, which is dependent on the IFNAR, but not on the TLR7-mediated MAPK p38 activation. Disturbed signaling causes IgG class-switch and secretion of the pro-inflammatory cytokine IL-6. Due to reduced inflammation, at least according to reduced serum IL-6 *in vivo* and *ex vivo*, cellular phenotypes of myeloid cells and B cell populations are partially restored in B6.*Sle1*.*yaa*.*Bank1*^*-/-*^ mice. Our results therefore show that *Bank1* deficiency may slightly ameliorate the development of clinical disease, but would require the combination of other genes to fully abrogate it.

## Supporting Information

S1 Fig GN scores of renal biopsies, and serum IL-6 and BUN measurements. (A) The severity index of cumulative GN in B6.*Sle1*.*yaa*.*Bank1* (*SY*.*Bank1*) mice at 20-24-wk of age was determined through PAS-stained slides. Pathological changes were scored by one observer blinded to the genotypes. Cumulative GN score is the summary of acute and chronic GN severity (mean; ns>0.05, Mann-Whiney nonparametric test). Total mice analyzed: *SY*.*Bank1*^+*/*+^ (n = 12), *SY*.*Bank1*^-*/*-^ (n = 10), and WT mice (n = 3). (B) BUN was measured in 24 weeks sera from *SY*.*Bank1*^*+/+*^ and *SY*.*Bank1*^*-/-*^ mice by using QuantiChrom Urea Assay Kit (BioAssay Systems). Each point represents value from one mouse and values were expressed in mg/dL. Each point represents value from one mouse, WT n = 3 mice, *SY*.*Bank1*^*+/+*^ n = 15 mice and *SY*.*Bank1*^*-/-*^ n = 11 mice. Bars show mean ±SD. p>0.05, not significant (ns). (C) Representative glomeruli from *SY*.*Bank1*^*+/+*^ and *SY*.*Bank1*^*-/-*^ mice at 21-wk stained with periodic acid-Schiff (PAS). Both strains showed enlarged hypercellular glomeruli with inflammatory cell infiltrates, although hyaline deposits are observed in the B6.*Sle1*.*yaa*.*Bank1*^*+/+*^ while the structure of the glomerulus is better kept in the B6.*Sle1*.*yaa*.*Bank1*^*-/-*^. 60X objective amplification was used. (D) Levels of serum IL-6 in 24 weeks old *SY*.*Bank1*^*+/+*^ (n = 17) and *SY*.*Bank1*^*-/-*^ (n = 12) mice as determined by ELISA. Each point represents the value from one mouse. Bars show mean ±SD. p>0.05, not significant (ns).
(JPG)

S2 Fig Flow cytometry analysis and gating strategies for different splenic B cell subsets and activation status in splenic B and T cells from B6.*Sle1*.*yaa*.*Bank1*^*+/+*^ and B6.*Sle1*.*yaa*.*Bank1*^*-/-*^ mice. (A) Representative FACS plots showed the gating strategies for marginal zone B (MZ B) and follicular B (FO B), transitional 1, 2, and 3 (T1, T2 and T3 B) B cells, CD23^-^IgM^lo/-^ immature B cells and B1a cells from total splenocytes. (B) The statistical data of the frequencies of T1, T2, T3 B and CD23^-^IgM^lo/-^ IM B cells are shown as percentage of total splenocytes. Total mice analyzed: *SY*.*Bank1*^*+/+*^ (n = 11), *SY*.*Bank1*^*-/-*^ (n = 13), WT (n = 8). Data pooled from 4 independent experimental cohorts of mice. Statistical plots are shown as mean with Mann-Whiney (*SY*.*Bank1*^*+/+*^ vs. *SY*.*Bank1*^*-/-*^) nonparametric test (*p≤0.05, ns>0.05). (C) Representative FACS plots showed the gating strategies for TCRβ^+^ T cells and CD19^+^ B cells from total splenocytes discriminated from debris and doublets, followed by examining percentages of CD69^+^ T and B cells.
(JPG)

S3 Fig Flow cytometry analysis and gating strategies for follicular helper T (T_fh_) cells and their surface expression of CXCR4 and CXCR5, germinal center (GC) B cells, and plasma cells from the splenocytes of B6.*Sle1*.*yaa*.*Bank1*^*+/+*^, B6.*Sle1*.*yaa*.*Bank1*^-/-^ and wild type B6 mice. (A) Representative FACS plots show the gating strategies for follicular helper T cells (T_fh_) from *SY*.*Bank1*^*+/+*^ and *SY*.*Bank1*^*-/-*^ mice. (B) Overlaid histogram plots demonstrate that CXCR4 expression on *SY*.*Bank1*^*-/-*^ T_fh_ cells is downregulated, compared with *SY*.*Bank1*^*+/+*^ T_fh_ cells. However, CXCR4 expression in T_fh_ cells is higher than that on CD19^+^ B cells. Filled grey histogram represents the isotype control for CXCR4. (C) Representative FACS plots show the gating strategies for germinal center B (GC B) cells. (D) Representative FACS plots show the gating strategies for plasma cells (PC). A-D, all quantified from total splenocytes discriminated from debris and doublets.
(JPG)

S4 Fig Flow cytometry analysis and gating strategies for immature B cells and mature recirculating B cells from the bone marrows of B6.*Sle1*.*yaa*.*Bank1*^+/+^, B6.*Sle1*.*yaa*.*Bank1* ^-/-^ and wild type B6 mice. Representative FACS plots show the gating strategies for immature B cells (IM B) and mature recirculating B cells quantified from bone marrows (BM).
(JPG)

S5 Fig Flow cytometry analysis and gating strategies for myeloid cells from spleen of B6.*Sle1*.*yaa*.*Bank1*^+/+^, B6.*Sle1*.*yaa*.*Bank1*^-/-^ and wild type B6 mice. (A) Representative FACS plots showing the gating strategies for CD11b^+^ myeloid cells quantified from total splenocytes. (B) Representative FACS plots showing the gating strategies for macrophages (gate I), Gr1^int^ monocytes (gate II), and neutrophils (gate III) from total splenocytes discriminated from debris and doublets.
(JPG)

S6 Fig Quantitative real-time PCR analysis of TLR7- and IFNAR-mediated gene expression. (A) Gene expression of *Irf5* and *Stat3* transcription factors was not modified upon R837 stimulation in *Bank1* deficient B cells. Purified splenic B cells were stimulated with TLR7 agonist (R837, 2 μg/ml) and gene expression was assessed with Taqman primers and probes. Expression was normalized to the 18s rRNA control gene. Results are representative of two-independent experiments. (B) Bank1 is not involved in the induction of gene expression through IFNAR signaling. Purified splenic B cells stimulated with rIFNα (2,000 U/ml) for the indicated times. None of the genes showed differences in expression in *Bank1* deficient B cells. (C) Expression of *Aicda* is not induced following rIFNα stimulation. RT-PCR of *Aicda* was done as in (A).
(JPG)

S7 Fig MAPK and NF-κB activation are comparable between B6.*Sle1*.*yaa*.*Bank1*^+/+^ and B6.*Sle1*.*yaa*.*Bank1*^-/-^ mice in response to TLR7 agonists. Purified B cells from *SY*.*Bank1*^*+/+*^ and *SY*.*Bank1*^*-/-*^ mice were stimulated with R848 (1 μg/ml) for the indicated periods and analyzed by immunoblotting with (A) phospho-p38, phospho-Erk1/2, total p38 and total Erk1/2 antibodies, and (B) phospho-Jnk, phospho-IκBα, Jnk and IκBα antibodies. Gapdh protein was used as loading control. Blots are representative of 3 independent experiments.
(JPG)

S8 Fig The impact of *Bank1* deficiency on activation of the Mnk1/2-eIF4E-mediated translation initiation pathway induced by type I IFN. (A) Activation of p38 following rIFNα stimulation (2000 U/ml). (B) Phosphorylation of Mnk1/2 following rIFNα (2000 U/ml) stimulation. (C) Phosphorylation of eIF4E following rIFNα stimulation. Band intensities of phospho-p38, phospho-Mnk1/2 and phospho-eIF4E relative to total p38, Mnk1/2 or eIF4E are shown beside each blot. Data are representative of three independent experiments. Differences were not significant except for the 15 minutes time point in activation of Mnk1/2, reduced in the *SY*.*Bank1*^*-/-*^ mice.
(JPG)
