# Supplementary figures and images for: BANK1 Regulates IgG Production in a Lupus Model by Controlling TLR7-Dependent STAT1 Activation
Source: PLoS One. 2016 May 26;11(5):e0156302. doi: 10.1371/journal.pone.0156302 (PMC4882053; doi:10.1371/journal.pone.0156302)

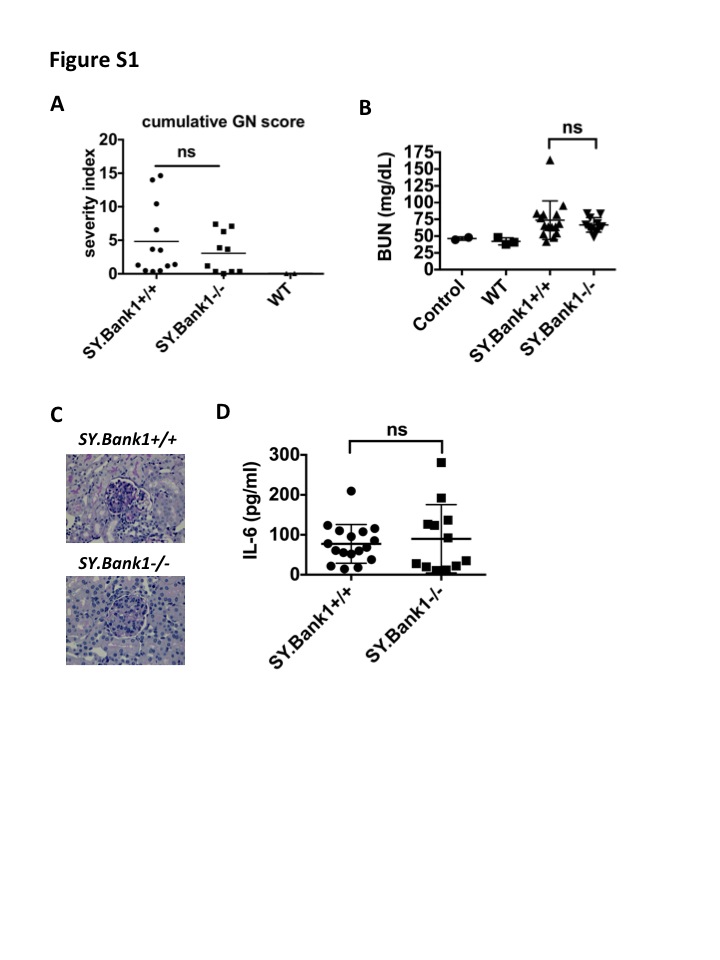

Supplement: S1 Fig — (A) The severity index of cumulative GN in B6.Sle1.yaa.Bank1 (SY.Bank1) mice at 20-24-wk of age was determined through PAS-stained slides. Pathological changes were scored by one observer blinded to the genotypes. Cumulative GN score is the summary of acute and chronic GN severity (mean; ns>0.05, Mann-Whiney nonparametric test). Total mice analyzed: SY.Bank1+/+ (n = 12), SY.Bank1-/- (n = 10), and WT mice (n = 3). (B) BUN was measured in 24 weeks sera from SY.Bank1+/+ and SY.Bank1-/- mice by using QuantiChrom Urea Assay Kit (BioAssay Systems). Each point represents value from one mouse and values were expressed in mg/dL. Each point represents value from one mouse, WT n = 3 mice, SY.Bank1+/+ n = 15 mice and SY.Bank1-/- n = 11 mice. Bars show mean ±SD. p>0.05, not significant (ns). (C) Representative glomeruli from SY.Bank1+/+ and SY.Bank1-/- mice at 21-wk stained with periodic acid-Schiff (PAS). Both strains showed enlarged hypercellular glomeruli with inflammatory cell infiltrates, although hyaline deposits are observed in the B6.Sle1.yaa.Bank1+/+ while the structure of the glomerulus is better kept in the B6.Sle1.yaa.Bank1-/-. 60X objective amplification was used. (D) Levels of serum IL-6 in 24 weeks old SY.Bank1+/+ (n = 17) and SY.Bank1-/- (n = 12) mice as determined by ELISA. Each point represents the value from one mouse. Bars show mean ±SD. p>0.05, not significant (ns). (JPG) [file pone.0156302.s001.jpg]

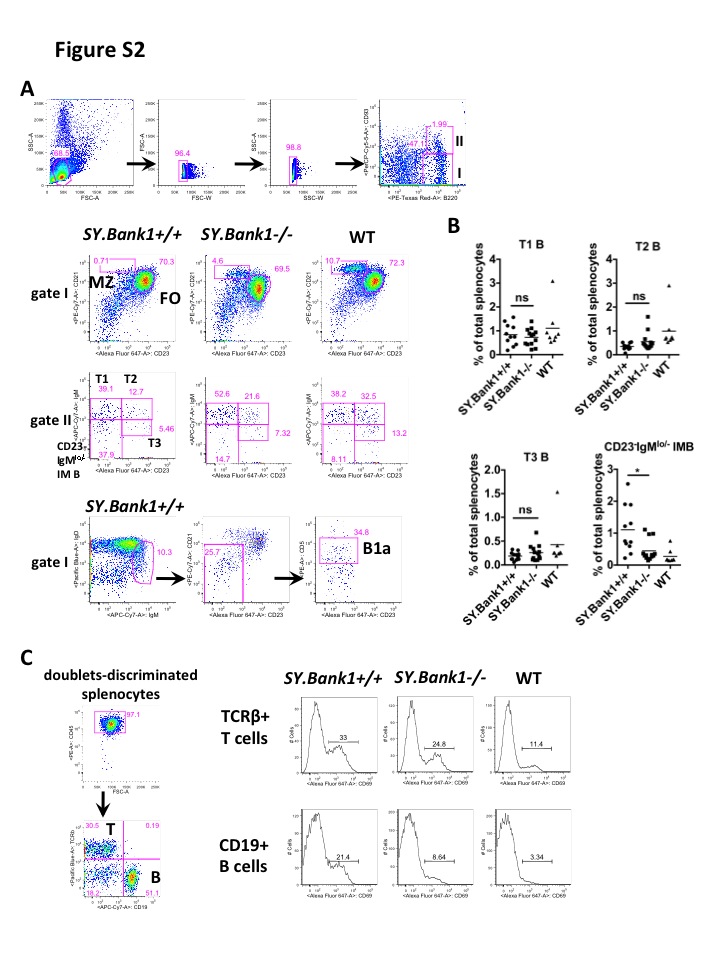

Supplement: S2 Fig — (A) Representative FACS plots showed the gating strategies for marginal zone B (MZ B) and follicular B (FO B), transitional 1, 2, and 3 (T1, T2 and T3 B) B cells, CD23-IgMlo/- immature B cells and B1a cells from total splenocytes. (B) The statistical data of the frequencies of T1, T2, T3 B and CD23-IgMlo/- IM B cells are shown as percentage of total splenocytes. Total mice analyzed: SY.Bank1+/+ (n = 11), SY.Bank1-/- (n = 13), WT (n = 8). Data pooled from 4 independent experimental cohorts of mice. Statistical plots are shown as mean with Mann-Whiney (SY.Bank1+/+ vs. SY.Bank1-/-) nonparametric test (*p≤0.05, ns>0.05). (C) Representative FACS plots showed the gating strategies for TCRβ+ T cells and CD19+ B cells from total splenocytes discriminated from debris and doublets, followed by examining percentages of CD69+ T and B cells. (JPG) [file pone.0156302.s002.jpg]

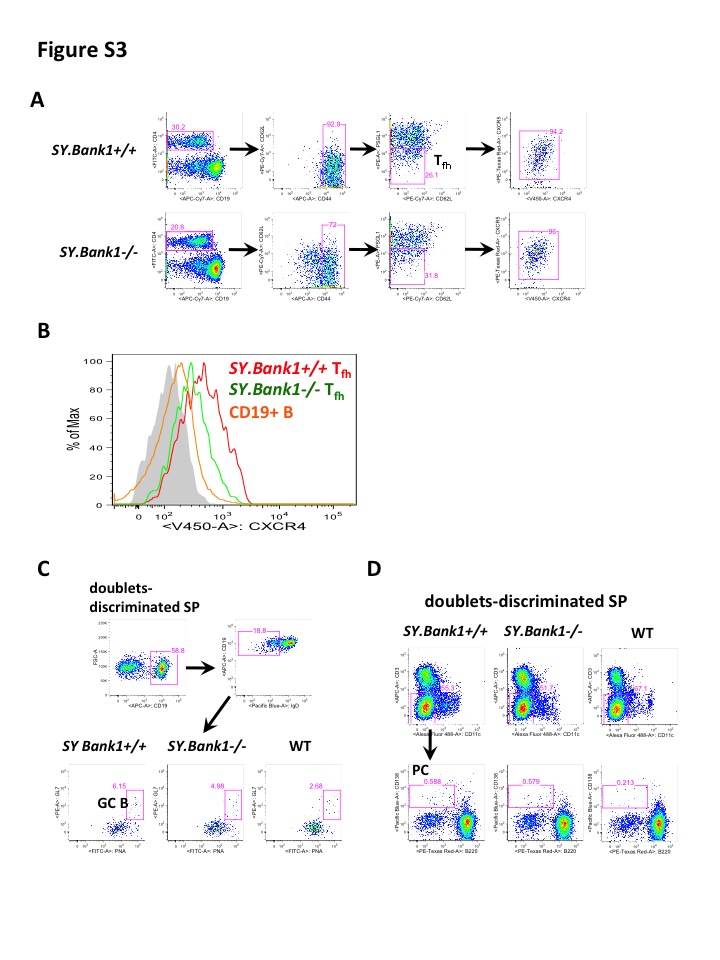

Supplement: S3 Fig — (A) Representative FACS plots show the gating strategies for follicular helper T cells (Tfh) from SY.Bank1+/+ and SY.Bank1-/- mice. (B) Overlaid histogram plots demonstrate that CXCR4 expression on SY.Bank1-/- Tfh cells is downregulated, compared with SY.Bank1+/+ Tfh cells. However, CXCR4 expression in Tfh cells is higher than that on CD19+ B cells. Filled grey histogram represents the isotype control for CXCR4. (C) Representative FACS plots show the gating strategies for germinal center B (GC B) cells. (D) Representative FACS plots show the gating strategies for plasma cells (PC). A-D, all quantified from total splenocytes discriminated from debris and doublets. (JPG) [file pone.0156302.s003.jpg]

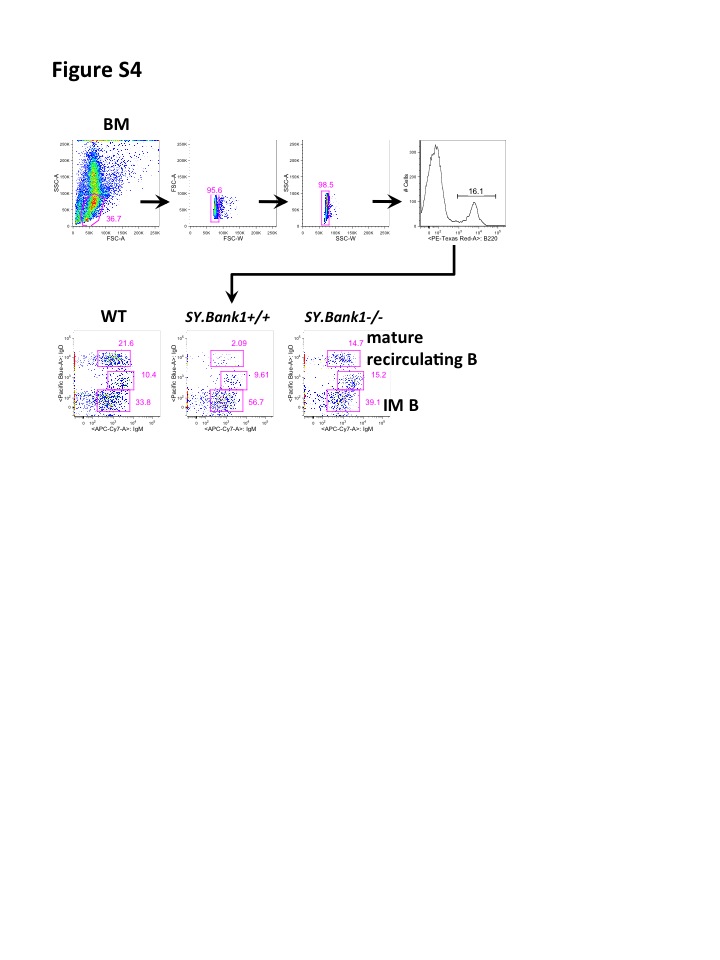

Supplement: S4 Fig — Representative FACS plots show the gating strategies for immature B cells (IM B) and mature recirculating B cells quantified from bone marrows (BM). (JPG) [file pone.0156302.s004.jpg]

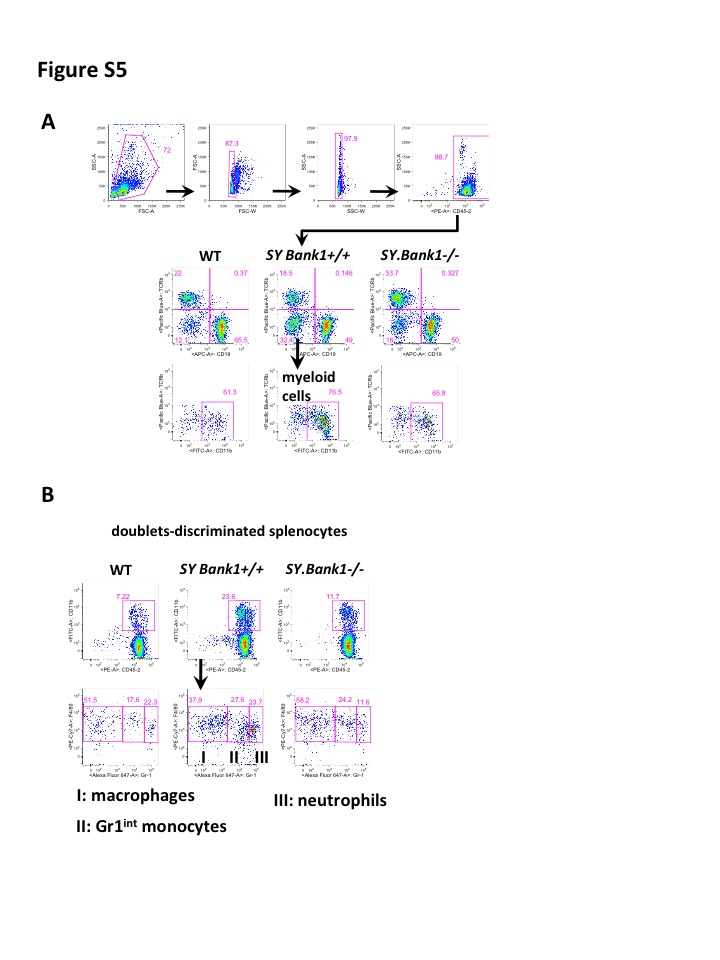

Supplement: S5 Fig — (A) Representative FACS plots showing the gating strategies for CD11b+ myeloid cells quantified from total splenocytes. (B) Representative FACS plots showing the gating strategies for macrophages (gate I), Gr1int monocytes (gate II), and neutrophils (gate III) from total splenocytes discriminated from debris and doublets. (JPG) [file pone.0156302.s005.jpg]

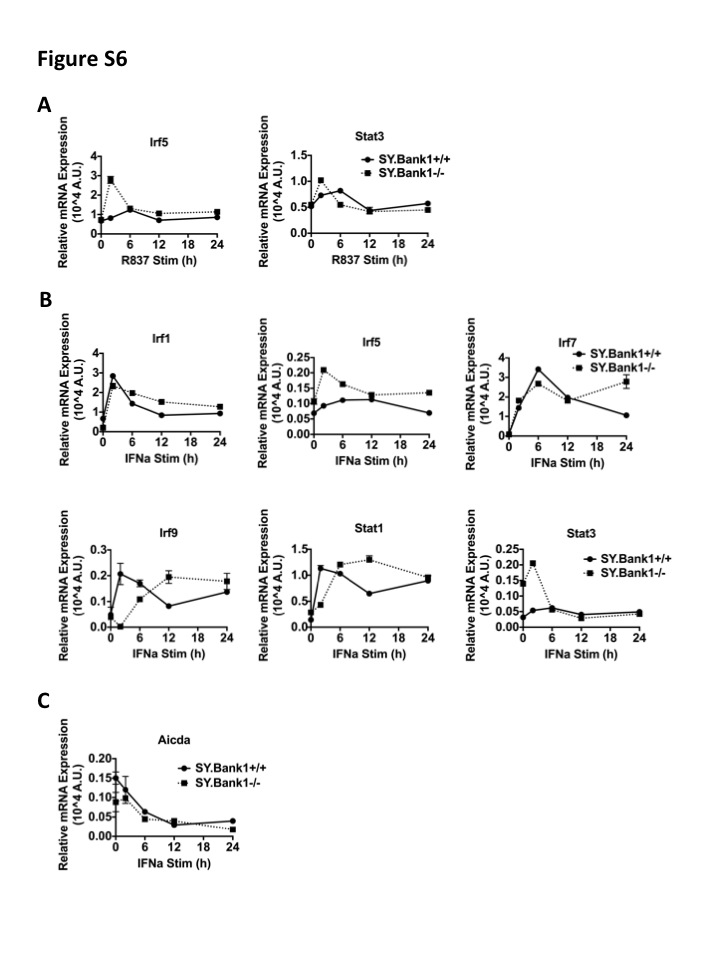

Supplement: S6 Fig — (A) Gene expression of Irf5 and Stat3 transcription factors was not modified upon R837 stimulation in Bank1 deficient B cells. Purified splenic B cells were stimulated with TLR7 agonist (R837, 2 μg/ml) and gene expression was assessed with Taqman primers and probes. Expression was normalized to the 18s rRNA control gene. Results are representative of two-independent experiments. (B) Bank1 is not involved in the induction of gene expression through IFNAR signaling. Purified splenic B cells stimulated with rIFNα (2,000 U/ml) for the indicated times. None of the genes showed differences in expression in Bank1 deficient B cells. (C) Expression of Aicda is not induced following rIFNα stimulation. RT-PCR of Aicda was done as in (A). (JPG) [file pone.0156302.s006.jpg]

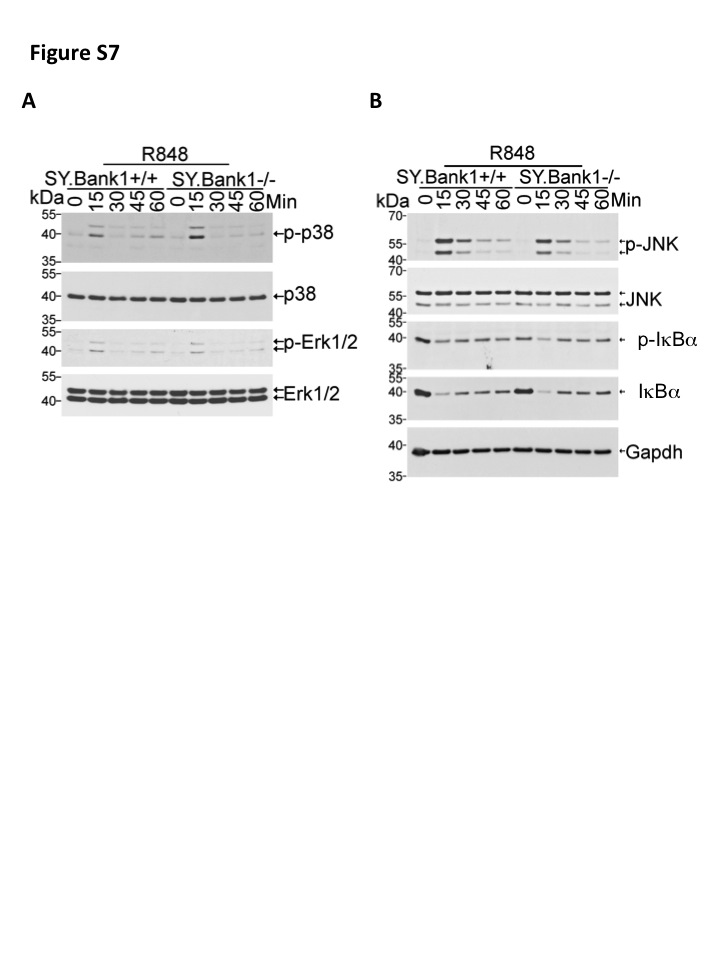

Supplement: S7 Fig — Purified B cells from SY.Bank1+/+ and SY.Bank1-/- mice were stimulated with R848 (1 μg/ml) for the indicated periods and analyzed by immunoblotting with (A) phospho-p38, phospho-Erk1/2, total p38 and total Erk1/2 antibodies, and (B) phospho-Jnk, phospho-IκBα, Jnk and IκBα antibodies. Gapdh protein was used as loading control. Blots are representative of 3 independent experiments. (JPG) [file pone.0156302.s007.jpg]

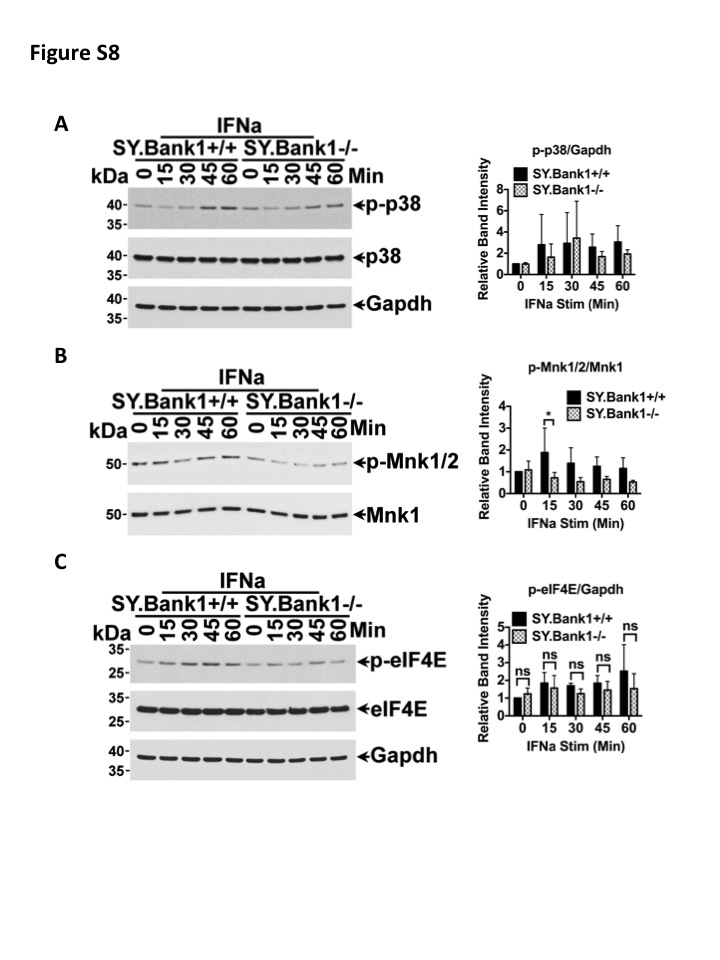

Supplement: S8 Fig — (A) Activation of p38 following rIFNα stimulation (2000 U/ml). (B) Phosphorylation of Mnk1/2 following rIFNα (2000 U/ml) stimulation. (C) Phosphorylation of eIF4E following rIFNα stimulation. Band intensities of phospho-p38, phospho-Mnk1/2 and phospho-eIF4E relative to total p38, Mnk1/2 or eIF4E are shown beside each blot. Data are representative of three independent experiments. Differences were not significant except for the 15 minutes time point in activation of Mnk1/2, reduced in the SY.Bank1-/- mice. (JPG) [file pone.0156302.s008.jpg]
